# In-depth amino acid mutational analysis of the key interspecific incompatibility factor Stigmatic Privacy 1

**DOI:** 10.1093/pcp/pcaf039

**Published:** 2025-04-15

**Authors:** Yoshinobu Kato, Shun Tadokoro, Shota Ishida, Maki Niidome, Yuka Kimura, Seiji Takayama, Sota Fujii

**Affiliations:** Graduate School of Agricultural and Life Sciences, The University of Tokyo, 1-1-1 Yayoi, Bukyo-ku, Tokyo 113-8657, Japan; Japan Science and Technology Agency, Precursory Research for Embryonic Science and Technology, 4-1-8 Honcho, Kawaguchi, Saitama 332-0012, Japan; Graduate School of Agricultural and Life Sciences, The University of Tokyo, 1-1-1 Yayoi, Bukyo-ku, Tokyo 113-8657, Japan; Graduate School of Agricultural and Life Sciences, The University of Tokyo, 1-1-1 Yayoi, Bukyo-ku, Tokyo 113-8657, Japan; Graduate School of Agricultural and Life Sciences, The University of Tokyo, 1-1-1 Yayoi, Bukyo-ku, Tokyo 113-8657, Japan; Graduate School of Agricultural and Life Sciences, The University of Tokyo, 1-1-1 Yayoi, Bukyo-ku, Tokyo 113-8657, Japan; Graduate School of Agricultural and Life Sciences, The University of Tokyo, 1-1-1 Yayoi, Bukyo-ku, Tokyo 113-8657, Japan; Graduate School of Agricultural and Life Sciences, The University of Tokyo, 1-1-1 Yayoi, Bukyo-ku, Tokyo 113-8657, Japan; Suntory Rising Stars Encouragement Program in Life Sciences (SunRiSE), 8-1-1 Seikadai, Seikacho, Kyoto 619-0284, Japan

**Keywords:** alanine and glycine scanning, *Arabidopsis thaliana*, interspecific incompatibility, STIGMATIC PRIVACY 1, *Malcolmia littorea*, multimeric protein complex

## Abstract

In plants, there is an active prezygotic interspecific-incompatibility mechanism to prevent unfavorable hybrids between two species. We previously reported that an uncharacterized protein with four-transmembrane domains, named as Stigmatic Privacy 1 (SPRI1), is responsible for rejecting heterospecific pollen grains in *Arabidopsis thaliana*. However, the lack of notable functional domains in SPRI1 has limited our understanding of its biochemical properties. In this study, we conducted a functional analysis of the SPRI1 protein through point-mutational experiments and biochemical analysis. We explored the molecular regulatory mechanisms of SPRI1 and the relationships with its function. Alanine- and glycine-scanning experiments together with the evolutional analysis showed that the structural integrity of the C-terminal regions of the extracellular domain of this protein is important for its function. In addition, we found two cysteines (C67 and C80) within the extracellular domain that may be involved in the formation of intermolecular disulfide bonds. These cysteine residues are required for the stabilization of the SPR1 protein. Furthermore, SPRI1 may form homo-multimers and is present as part of a ∼300 kDa complex. Our present study indicates that SPRI1 forms large protein machinery for the rejection of hetero-specific pollen in stigmatic papilla cells.

## Introduction

Pre-zygotic reproductive barrier is an important biological system that prevents formation of unfavorable interspecific hybrids (de Nettancourt 2001). In plants, such reproductive barriers can be found in the process of inter-cellular communication between the male gametophyte pollen and the female pistil tissues (Tsuchimatsu and Fujii 2022). Recently, we found a stigmatic protein called Stigmatic Privacy 1 (SPRI1) (Fujii et al. 2019), as the key factor for regulating heterospecific pollen rejection in *Arabidopsis thaliana*. SPRI1 is a plasma-membrane localized protein with four-transmembrane domains. SPRI1 functions to reject pollen grains from other Brassicaceae species, and heterospecific pollen tubes may grow into the mutants of SPRI1 while they are unable to do so in the wild-type stigma. It has been considered that prezygotic reproductive barrier can be broken down into incongruity and incompatibility. An incongruity arises due to the passive loss of gene functions involved in the fertilization process, probably acting as a driver of reproductive isolation during speciation. A pair of interactors, such as pistil cysteine-rich protein LURE1 and the cognate pollen-side receptor PRK6, has been known as the example of inter-specific incongruity causing factors in plant reproduction (Takeuchi and Higashiyama 2016). On the other hand, SPRI1 is the first-reported interspecific incompatibility causing factor that actively rejects heterospecific pollen (Fujii et al. 2019). More recently, some factors involved in forming interspecific reproductive barriers. SPRI2 and its SPRI2-like variant belong to the SHORT-INTERNODES (SHI) transcription factor family and may modulate the expression of cell wall modification genes as well as SPRI1 (Fujii et al. 2023). Additionally, FERONIA/CURVY1/ANJEA/HERCULES RECEPTOR KINASE 1 along with cell wall proteins LRX3/4/5 work in concert with RALF peptides or SRK to establish interspecific reproductive barriers (Huang et al. 2023, Lan et al. 2023). It has been reported that leucine-rich repeat malectin receptor-like kinases are also involved in the rejection of foreign pollen (Lee et al. 2024). Thus, SPRI1 is one of the few known factors that form the prezygotic reproductive barrier through the incompatibility function. Therefore, there is great anticipation to uncover its cellular role.

Due to the lack of notable functional protein domains in SPRI1, its molecular role remains unclear. SPRI1 consists of four-transmembrane domains, two putative extracellular, and three intracellular regions. The four-transmembrane-based secondary structure of SPRI1 resembles that of the periplasmic protein DsbB in *Escherichia coli* (Inaba and Ito 2008). DsbB forms a disulfide bond in its periplasmic regions and acts as a redox potential transducer across the cytoplasmic membrane. The tetraspanins are another protein family ubiquitously found in eukaryotic cells with secondary structures similar to SPRI1 or DsbB. Tetraspanins play a diverse role in cells such as regulation of signaling, cell-to-cell adhesion or fusion, fertilization, and pathogen infection (Charrin et al. 2014). One of the proposed roles of the tetraspanins is to form a large molecular network in the membrane systems through direct or indirect protein–protein interactions, referred to as the tetraspanin ‘web’ in a previous study (Boucheix and Rubinstein 2001). In addition, there are some proteins which consist of four-transmembrane domains and form oligomeric structures to function as ion or gap junction channels (Burendei et al., 2020, Collingridge et al. 2009).

In this study, we aimed to understand the biochemical function of SPRI1. In analogy with DsbB or tetraspanins, we suspected that the first 36 amino-acid-long extracellular regions in SPRI1 may play a critical role, and we performed a screening via point mutations. We show that disulfide bonds and complex formation abilities of SPRI1 are critical for its function to express interspecific incompatibility.

## Results

### Amino acid substitutions in the long extracellular domain of SPRI1 highlights amino acids required for its stability

**Figure 1.**
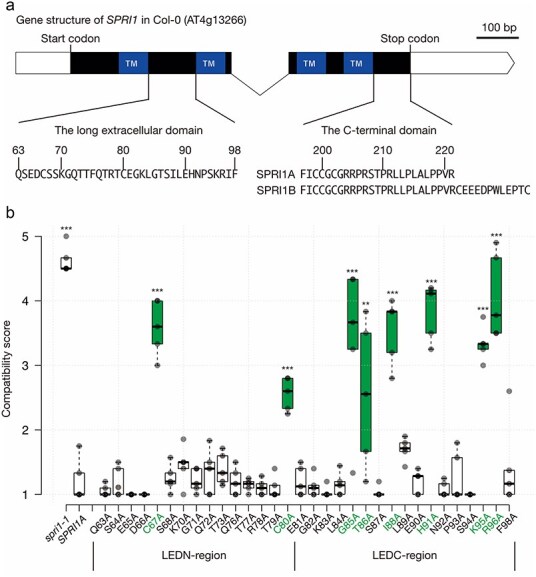
Gene structure of SPRI1 and alanine replacement analysis of the long extracellular domain. (a) Gene structure of SPRI1. TM: transmembrane region. (b) Summary of the interspecific pollination analysis of the transformants introduced with the alanine-replacement mutant SPRI1 forms. Significant differences by Dunnett’s test compared against *SPRI1A* (*spri1* mutant complemented with the nonmutated version of *SPR1A*) are indicated by **(*P *< .01) and ***(*P* < .005). The *n* = 5 independent transgenic lines for the point mutation transformants. The *n* = 5 individuals for *spri1-1* and *SPRI1A* line.

The SPRI1 protein contains two putative extracellular domains based on its anticipated membrane insertion topology (Figure S1A). We paid attention to the longer domain, which is predicted to span from the 63rd glutamine (Q63, amino acid residues are abbreviated in this manner hereafter) to F98 (Fig. 1a). To understand the role for each of these residues, we substituted 32 of these amino acid residues by alanine and introduced these mutant SPRI1A (functional allele of SPRI1 found in the previous study) forms into the *spri1-1* mutant, in which *SPRI1* gene expression was below the detection limit in RT-qPCR due to a T-DNA insertion in the 5ʹUTR of the *SPRI1* gene (Fujii et al. 2019). Alanine was used because it is non-bulky and chemically inert (Morrison and Weiss 2001). We excluded the amino acids that were not conserved in the SPRI1 sequence of *Arabidopsis lyrata* from the analysis. In our previous study, we confirmed that SPRI1 in *A. lyrata* is functional (Fujii et al. 2019), and we considered the amino acids not conserved between *A. thaliana* and *A. lyrata* as non-essential for its function (Figure S1B). The lines expressing the alanine-replaced SPRI1A forms were pollinated with the pollen grains of *Malcolmia littorea*, a species belonging to the Brassicaceae family whose pollen has been shown to be rejected by the function of SPRI1 in our previous study (Fujii et al. 2019). As a result, we found that replacements of amino acids C67, C80, G85, T86, I88, H91, K95, and R96 have caused significant impairment of the function of SPRI1 *in vivo* (Fig. 1b).

### Structural stability at the evolutionary conserved region in the long extracellular domain is important for SPRI1 function

**Figure 2.**
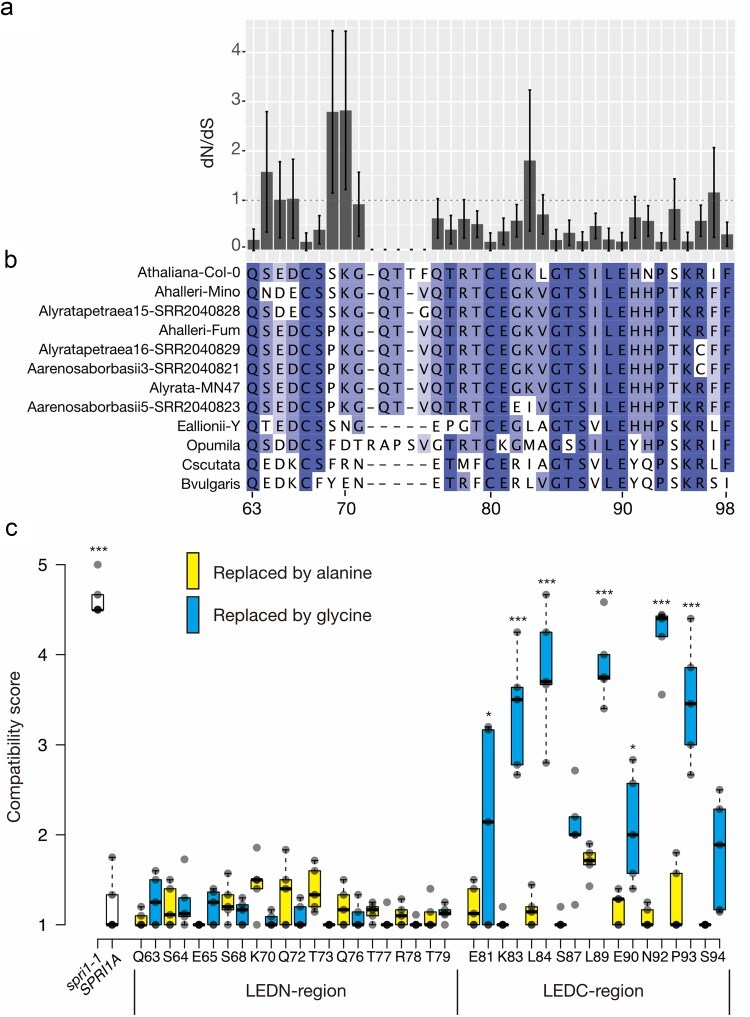
Evolutionary analysis of SPRI1 and glycine replacement analysis of the long extracellular domain. (a) Bargraph showing dN/dS calculated from the SPRI1 multiple alignments from 58 Brassicaceae species. (b) Amino acid sequence alignment of the long extracellular domain found from representative Brassicaceae species. (c) Summary of the interspecific pollination analysis of the transformants introduced with the alanine-replacement or the glycine-replacement mutant SPRI1 forms. Significant differences by Dunnett’s test compared against *SPRI1A* (*spri1* mutant complemented with the nonmutated version of *SPR1A*) are indicated by *P < .05 and ***P < .005. The *n* = 5 independent transgenic lines for the point mutation transformants. The *n* = 5 individuals for *spri1-1* and *SPRI1A* line.

From the abovementioned results, we noticed that replacements of the C-terminal half of the long extracellular domain (arbitrarily named as the LEDC-region, Fig. 1a) with alanine cause frequent impairment to the SPRI1 function. We reasoned that this could be due to a stronger evolutionary pressure to preserve the protein structure on the LEDC-region compared to the N-terminal region of the long extracellular domain (LEDN-region). Thus, we analyzed the sequences from 58 *SPRI1* orthologous genes collected in our previous study (Fujii et al. 2019), and calculated the ratio of non-synonymous to synonymous substitution rates (dN/dS) (Fig. 2a). We observed that the dN/dS ratio for amino acid positions in the LEDN-region frequently exceeded 1, indicating that these codons are either experiencing relaxed selective pressure or are under positive selection (Fig. 1a). We were unable to obtain dN/dS ratio for some residues due to amino acid insertion/deletions in this region, further implying that this LEDN-region could be diverged (Fig. 2b). In contrast, dN/dS ratio of most codons in the LEDC-region are found to be <1 (Fig. 2a), suggesting that they are more likely to be under negative selection and are structurally conserved.

Next, we asked if increasing protein structural flexibilities in these regions would indeed impair the SPRI1 function. We selectively replaced the amino acid positions that were able to be replaced by alanine without any functional compromises (Fig. 1b) with glycine, the smallest amino acid which renders largest conformational flexibility to a protein structure. Substitution with glycine sometimes causes different effects from those of alanine (Trenevska et al. 2021). As a result, while replacement with glycine in the LEDN-region did not exhibit any effect, most of the glycine replacements in the LEDC-region impair the molecular function of SPRI1A (Fig. 2c). Taken together with the evolutionary analysis, these data indicated that the C-terminal regions of the long extracellular domain are structurally important to maintain the function of SPRI1.

### Two cysteine residues in the long extracellular domain may be involved in forming the disulfide bonds

**Figure 3.**
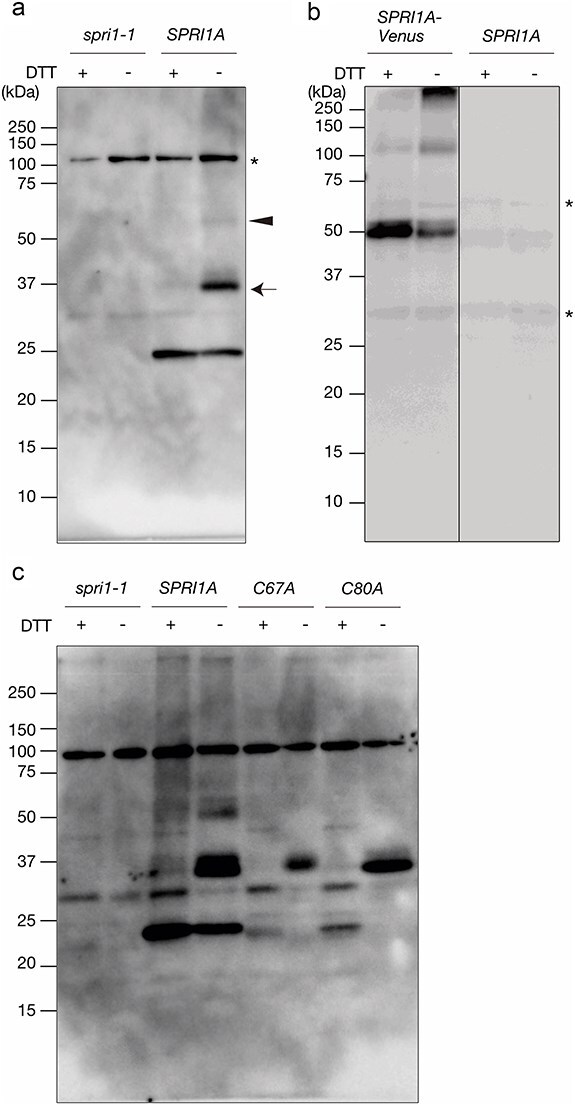
SPRI1A forms disulfide bonds. (a) Total membrane proteins were separated under the presence (+) or absence (−) of DTT conditions in SDS-PAGE followed by immunoblotting using the antibody against SPRI1. *spri1-1* was used as a negative control. A black arrow and an arrowhead indicate the putative SPRI1 dimer and some higher order structure, respectively. A black asterisk indicates nonspecific signal. (b and c) The same analysis as in Fig. 3a was performed using *SPRI1A–Venus* with the antibody against GFP in (b) and using *SPRI1A_C67A* (*C67A*) and *SPRI1A_C80A* (*C80A*) with the antibody against SPRI1 in (c). The uncropped image of (b) was shown in Figure S7.

We also found that the two cysteine residues C67 and C80 were the only indispensable amino acids in the LEDN-region (Fig. 1b). We suspected that these residues could be involved in intra- or inter-molecular disulfide bond formation. To investigate the possible involvement of disulfide bond formation in SPRI1, we performed immunoblots in the absence (−) or the presence of the reducing agent dithiothreitol (DTT). When treated with DTT, a single band was detected close to its expected monomeric molecular size 25.1 kDa (Fig. 3a). In contrast, in the absence of DTT, we detected an additional band close to the 37 kDa marker (indicated by the arrow) and a faint band above the 50 kDa marker (indicated by the arrowhead) (Fig. 3a). There are two possibilities to interpret this result: One is SPRI1 forms disulfide bonds with other protein(s), and another is SPRI1 forms inter-molecular disulfide bonds with itself. Because of the quite low accumulation of a faint band around 50 kDa, we consider this band is not a predominant form of SPRI1 and does not significantly contribute to the SPRI1 function. To verify above hypothesis, we developed a transgenic *spri1-1* line expressing the fusion protein of SPRI1A and a fluorescent protein Venus (Nagai et al. 2002). We reasoned that if SPRI1–SPRI1 disulfide bond is formed, the size shift of SPRI1A–Venus in the non-reducing condition will become greater because of the Venus tag fusion. If SPRI1 forms disulfide bonds with other protein(s), the band shift will not be affected by Venus fusion. We confirmed that this SPRI1A–Venus line can strongly reject pollen grains of *M. littorea* (Figure S2), indicating that the native function of SPRI1A is retained in this transgenic line. In both presence and absence of DTT, the SPRI1A–Venus signals detected by the anti-GFP antibody were found at around its expected monomeric molecular size 52.7 kDa (Fig. 3b). In the absence of DTT, an additional band at around >100 kDa was strongly detected compared to that in the presence of DTT (Fig. 3b). This size shift observed in the SPRI1A–Venus transgenic line (Fig. 3b: about 50 kDa) was greater than that in the native SPRI1A (Fig. 3a: about 10–15 kDa), supporting the idea that the higher molecular band (indicated by the arrow) in the non-reducing condition may be composed of SPRI1A-homodimer. The apparent molecular size shift in the native SPRI1A dimer was smaller than its expected size (Fig. 3a; 25 kDa + 25 kDa), probably due to the incomplete denaturation and compact packing under DTT-less condition. SDS-PAGE just shows the apparent molecular size and does frequently not reflect the true molecular size. A similar band shift pattern has been reported for CD9, which contains four transmembrane regions as seen in SPRI1. The estimated molecular size of CD9 is 25 kDa, but its disulfide bond-linked homodimer was observed as a 38 kDa band in non-reducing SDS-PAGE gels (Kovalenko et al. 2004).

To further investigate the involvement of C67 and C80 in the disulfide bond formations, we detected the molecular accumulation pattern of SPRI1A protein in the alanine-substitution lines of these two amino acids along with other mutations (H91A, K95A, or R96A). The accumulation of SPRI1 protein in the H91A, K95A, and R96A mutants was below the detection limit (Figure S3). On the other hand, plants expressing C67A and C80A accumulated significant amount of SPRI1 protein (Figure S3). The transcript levels of *H91A* and *R96A* were equivalent to the wild-type *SPRI1A*, and the level of *K95A* was similar to those of *C67A* and *C80A* (Figure S6A). This indicates that the gene expressions of H91A, K95A, and R96A were sufficient to detect in immunoblotting, but each alanine mutation affected protein stability. In non-reducing condition, only the putative dimer forms were detected in the C67A and C80A mutants (Fig. 3c). This may indicate that both C67–C67 and C80–C80 disulfide bonds could be formed. The slight mobility difference of the putative dimeric bands detected in the two mutants (Fig. 3c and S3) may reflect the difference of the molecular conformations in the C67–C67 (detected in C80A) and C80–C80 (detected in C67A) forms. These results suggested that SPRI1A could form inter-molecular disulfide bonds.

**Figure 4.**
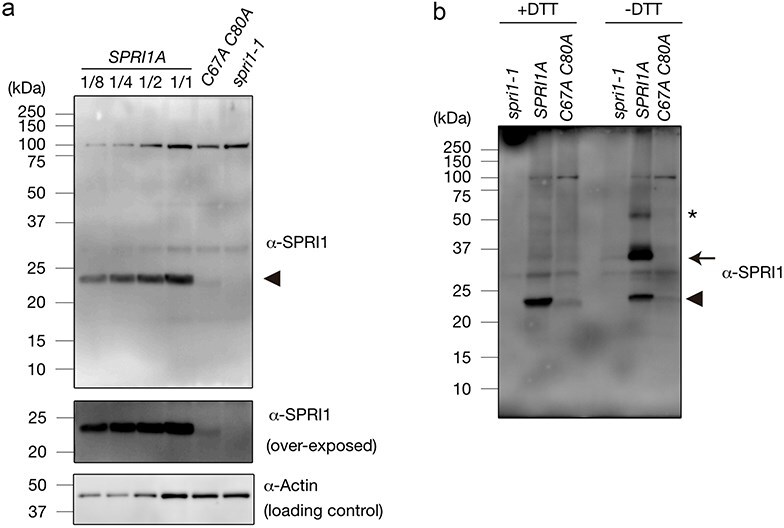
C67 and C80 are essential for the SPRI1 accumulation. (a) Total membrane proteins isolated from stigma samples of *SPRI1A, SPRI1A_C67A C80A* (*C67A C80A*), and *spri1-1* were analyzed by immunoblotting using antibody against SPRI1. Sample loading was based on the number of stigmas, along with a dilution series of *SPRI1A*. Actin in total soluble fractions was detected as a loading control using its specific antibody. (b) The same analysis as in Fig. 3a was performed using stigma samples of *spri1-1, SPRI1A*, and *SPRI1A_C67A C80A* plants. Black arrowheads and an arrow indicate the SPRI1 monomer and putative dimer, respectively. An asterisk indicates some higher-order structure.

On the other hand, SPRI1A monomer was not detectable under absence of DTT in these mutants, although it was stably accumulated in the plant expressing native SPRI1A (Fig. 3c). It is possible that an intra-molecular disulfide bond between C67 and C80 is also formed and contributes to the monomeric SPRI1 stability. To test this possibility, we produced a transgenic plant expressing SPRI1A of which both C67 and C80 were substituted to alanine and detected the SPRI1A protein in the transformants. In this transgenic plant, the accumulation level of the SPRI1A protein was drastically reduced to less than 1/8 compared to the wild-type SPRI1A (Fig. 4a). The transcript level of *SPRI1A_C67AC80A* was normally accumulated (Figure S6B). As opposed to the single point-mutated C67A or C87A plants, only the monomeric SPRI1A form was detected in the C67A_C80A plant under the non-reducing condition (Fig. 4b). Since this monomeric SPRI1A can stably accumulate in the wild-type plant, it is possible that intra-molecular disulfide bond between C67 and C80 make monomeric SPRI1A stable. This also explains why the severe reduction of monomeric SPRI1 in *C67A* or *C80A* plants is seen under the non-reducing SDS-PAGE condition (Fig. 3c), since the C67A or C80A mutation likely disrupts the intra-molecular disulfide bond formation between C67 and C80 making this protein less stable. Taken together, both intra- and inter-molecular disulfide bonds are formed via C67 and C80, and they are crucial for stabilizing SPRI1 monomer and dimer.

### SPRI1 is found within a high-molecular complex including its homomultimer forms

**Figure 5.**
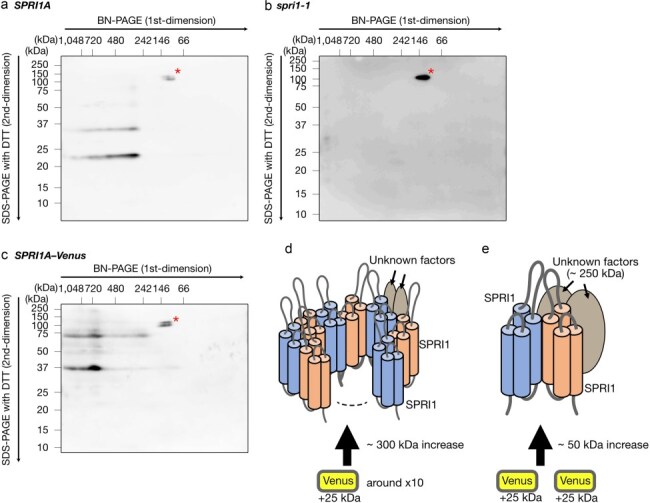
SPRI1A forms a protein complex of which size is about 300 kDa. (a–c) Stigmatic membrane protein complexes derived from *SPRI1A* (a), *spri1-1* (b), and *SPRI1A–Venus* (c) were separated by BN-PAGE and further subjected to two-dimensional SDS-PAGE under the presence of DTT condition. The SPRI1 protein was detected using the antibody against SPRI1. Asterisks indicate nonspecific signals. It was observed that the migration of the SPRI1A-Venus monomer and putative SPRI1A-Venus dimer in two-dimensional SDS-PAGE was faster than in standard SDS-PAGE (Fig. 3b). This was probably due to the Venus moiety being undenatured without TCA treatment, which cannot be applied to a BN-gel strip. (d and e) Schematic of possible SPRI1 complex models. SPRI1 is indicated in blue and orange, while other unknown factors within the complex are indicated in brown. In (d), the SPRI1 multimer is the major component in this model. Attachment of multiple Venus molecules to the SPRI1 multimer results in a significant enlargement of the SPRI1 complex, consistent with the observation in (a and c), where a large difference in molecular complex size was found between SPRI1 and SPRI1-Venus. Note that this model does not indicate specific stoichiometry of the SPRI1 complex but merely show the homopolymeric state. In (e), the major component(s) of the SPRI1 complex in this model is the unknown factor(s) that interacts with SPRI1. However, attachment of only a few Venus molecules causes only a slight increase in the SPRI1 complex, which disagrees with the observation in (a and c).

We next suspected that SPRI1 could reside in a higher-order complex. We therefore used the Blue Native-PAGE (BN-PAGE) to study the native SPRI1 protein complex, and the possible recruitment of SPRI1 homopolymers therein. We used the mild detergent *n*-dodecyl-β-d-maltoside to solubilize the SPRI1 complex from the membranes in the homogenized stigmatic cells and subjected to the two-dimensional BN/SDS-PAGE. As a result, SPRI1 was detected at the size between 242 and 480 kDa in the BN-gel (Fig. 5a). These signals were not detected in the *spri1-1* mutant (Fig. 5b), indicating that they are specific signals of SPRI1A. The SPRI1 monomer and the putative homodimers were detected even in the DTT-treated BN-gel strip, because not enough amount of DTT could reach the SPRI1 proteins in the gel strip during the SDS sample buffer treatment. In contrast, we observed SPRI1 signals near 720 kDa in the SPRI1–Venus expressing line under the same BN-PAGE conditions (Fig. 5c). This finding suggests that the putative SPRI1 complex increased in size substantially by fusing Venus to SPRI1 monomers. This result implies that the high-order SPRI1-multimer likely constitute the primary component of this complex (Fig. 5d), rather than the possibility that proteins other than SPRI1 occupy majority this complex (Fig. 5e).

**Figure 6.**
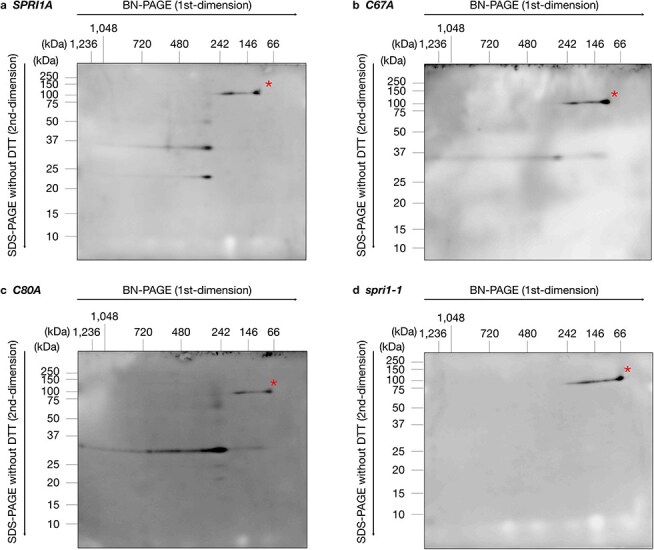
The SPRI1 protein complex contains both intra- and inter-molecular linked SPRI1. (a–d) Stigmatic membrane protein complexes derived from *SPRI1A* (a), *SPRI1A_C67A* (b), *SPRI1A_C80A* (c), and *spri1-1* (d) were separated by BN-PAGE and further subjected to two-dimensional SDS-PAGE under the absence of DTT condition. The SPRI1 protein was detected using the antibody against SPRI1. Asterisks indicate nonspecific signals.

In addition, SPRI1 was detected in the absence of DTT in two-dimensional SDS-PAGE (Fig. 6). The putative SPRI1 protein complex (∼300 kDa) exhibited various redox states of SPRI1A, including intra- and inter-molecular disulfide bonds (Fig. 6a). This observation suggests that the SPRI1 homo-oligomer is a mixture of SPRI1 monomers, putative homodimers, and higher molecular structures linked by disulfide bonds. The putative SPRI1 protein complex (∼300 kDa) was also present in the C67A and C80A mutants (Fig. 6b, c), but it consisted purely of SPRI1 inter-molecular dimers. This indicates that the formation of the SPRI1-multimers is not dependent on specific disulfide-bond structures, and the SPRI1-multimer is composed of even number of SPRI1.

To further confirm the SPRI1-multimeric structure, we transiently expressed *SPRI1A-Venus* and *SPRI1A-2xHA* in *Nicotiana benthamiana* leaves and performed immunoprecipitation using the antibody against GFP (Figure S4). SPRI1A–Venus was concentrated through this immunoprecipitation. As these samples were not treated with TCA, Venus was not fully denatured, and the mobility of SPRI1A–Venus during SDS-PAGE was faster compared to that in Fig. 3b. Whereas SPRI1A-2xHA was not detected in the absence of *SPRI1A-Venus* expression, both the dimeric and monomeric forms of SPRI1A-2xHA were co-immunoprecipitated with SPRI1A-Venus (Figure S4). This result further supports the idea that SPRI1 can form multimers, comprising both its monomeric and dimeric structures.

### C-terminal sequences and putative palmitoylation sites are also required for the function of SPRI1

**Figure 7.**
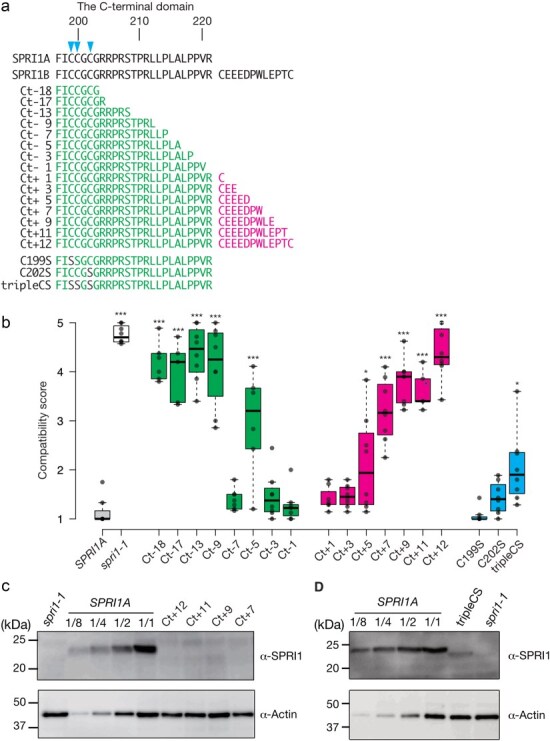
The intact C-terminal sequence is essential for the SPRI1 function. (a) Diagram showing the C-terminal region of SPRI1 and the deletion or the extension mutant series. Arrow heads indicate the cysteine residues which were predicted as palmitoylation sites. (b) Summary of the interspecific pollination analysis of the transformants introduced with the C-terminal deletion series (Ct-18 to Ct−1), the extension series (Ct+1 to Ct+12) or the putative palmitoylation sites. Significant differences by Dunnett’s test compared against *SPRI1A* (*spri1* mutant complemented with the non-mutated version of *SPR1A*) are indicated by *(*P *< .05) and ***(*P* < .005). Then *n* = 5–8 independent transgenic lines for the mutation introduced transformants. The *n* = 8 individuals for *spri1-1* and *SPRI1A* line. (c and d) Total membrane proteins isolated from stigma samples of *SPRI1A, spri1-1*, and a series of C-terminal extension lines (Ct+12, Ct+11, Ct+9, and Ct+7) in (c) or tripleCS in (d) were analyzed by immunoblotting using antibody against SPRI1. Sample loading was based on the number of stigmas, along with a dilution series of *SPRI1A*. Using total soluble proteins, actin was detected as a loading control with its specific antibody.

Apart from the long extracellular domain, we also reinvestigated the function of the C-terminal domain in this study. In our previous works, we found that some *Arabidopsis* natural strains (haplotype *SPRI1B* carriers) had lost their SPRI1 function due to the addition extra 12-amino acids at the C-terminus caused by a frameshift mutation (Fujii et al. 2019) (Fig. 7a). We analyzed the accumulation of SPRI1 protein in Kni-1, which is a natural strain expressing *SPRI1B* (Fujii et al. 2019). The accumulation level was below the detection limit in Kni-1 (Figure S5), suggesting that the SPRI1B stability was quite low. In the previous study, we showed that addition of this C-terminus extension to the functional SPRI1A can compromise its function, leading to the idea that this region is functionally important (Fujii et al. 2019). To further investigate the regulation of SPRI1A by the C-terminal region, we created a series of deletion and extension lines (Fig. 7a) and introduced these mutant SPRI1A forms into the *spri1-1* mutant. We found that deletion of 9 amino acids, or extension of 7 amino acids at the C-terminus of SPRI1 caused severe defects to its function (Fig. 7b). In the deletion series of C-terminus of SPRI1A, Ct-5 caused the impairment of SPRI1 function, although Ct-3 and Ct-7 lines showed normal heterospecific pollen rejection (Fig. 7b).Since the transcript level of *SPRI1* in Ct-5 was comparable to the *SPRI1A* plant (Figure S6D), the C-terminus of Ct-5 might abnormally affect the SPRI1 function or accumulation. In some of the extension lines (Ct+7, Ct+9, Ct+11, Ct+12), we found that SPRI1 protein accumulation level was below the detection limit, although SPRI1A fused with Venus at its C-terminus can fully complement its function accumulation (Figure S2A). It was confirmed that the transcript level of *SPRI1A* in Ct+7 and Ct+11 was comparable or higher than the *SPRI1A* plant (Figure S6C). Thus, it was possible that some specific amino acid sequence in the C-terminal extension can cause protein instability (Fig. 7c).

In tetraspanin proteins such as CD9, post-translational palmitoylation of the juxtamembrane cysteine residues plays an important role in the protein–protein interactions (Charrin et al. 2002). Since juxtamembrane cysteine residues at the 199, 200, and 202 positions of SPRI1A were strongly predicted as the candidate palmitoylation sites (Fig. 7a) using CSS-Palm 4.0 (Ren et al. 2008), we replaced these amino acids with serine to investigate their contributions to the SPRI1A function. As a result, although single replacements (C199S, C202S) did not significantly affect the SPRI1A function, mutant of all three sites (tripleCS) was partially but significantly impaired in the SPRI1A (Fig. 7b). The accumulation level of the SPRI1 protein was affected in tripleCS (Fig. 7d), whereas its transcript level was comparable to the *SPRI1A* plant (Figure S6D). In addition, the mobility during SDS-PAGE became slightly higher compared to the wild-type SPRI1A. As palmitoylation causes a slight reduction in mobility in SDS-PAGE (Gorleku et al. 2011), the increased mobility of SPRI1A_tripleCS might reflect a loss of its putative palmitoylation sites. These results suggested that the C-terminal intracellular flexible region of SPRI1A is important for its stability, as well as the putative palmitoylation sites.

## Discussion

In this study, we used mutant analysis to investigate the molecular nature of the SPRI1 protein. In the long extracellular region, we found that there is an evolutionary conserved subregion (LEDC-region) required for the function of SPRI1 (Figs. 1 and 2). Specifically, almost all the amino acids in LEDC region were not replaceable with glycine (Fig. 2), indicating that structural stability of this region is important for SPRI1 function. We also found that length of the flexible C-terminus regions is also a critical factor for the interspecific pollen rejection ability of SPRI1 (Fig. 7). These regions may be required to stabilize the SPRI1 molecule *in vivo* because we failed to detect detectable amount of SPRI1 protein in many of the mutants in these regions (Fig. 7c and S3).

We also discovered that two cysteine residues within the long extracellular region are crucial for SPRI1 function in the stigma (Fig. 1). It was possible that cysteine residues participate in the formation of intra- and inter-molecular disulfide bonds (C67–C67 and C80–C80) of SPRI1 in the stigma (Figs. 3 and 4). We found that the C67A mutation had a more severe effect on the interspecific incompatibility phenotype compared to the C80A mutation (Fig. 1b). This may be correlated with the observation that C67–C67 bridged inter-molecular dimer form of SPRI1 may be relatively more stable than the C80–C80 mediated form (Figure S3), indicating that C67–C67 plays a more significant role in the stability of SPRI1 in cells. Our observation also suggests the formation of intra-molecular C67–C80 disulfide bonds, as the monomeric form was absent in the C67A and C80A mutants, while it was abundant in the wild-type (Fig. 3c). Since impairment of interspecific incompatibility was observed in the C80A plant, where the total amount of SPRI1 was unchanged (Fig. 1 and S3), it is likely that C67–C80 intra- and/or inter-molecular disulfide bond is required for the full function of SPRI1. Recently, crystal structure of the four-transmembrane tetraspanin protein CD9 was revealed (Umeda et al. 2019, 2020). CD9 was found to form two intra-molecular disulfide bonds via four well-conserved cysteine residues in its large extracellular region, and these bridges were considered as important to stabilize this region (Umeda et al. 2020). It is possible that similar intra-molecular disulfide bond mechanism stabilizes the SPRI1 extracellular region. Regarding tetraspanins, palmitoylation of the cysteines at the end of transmembrane helices of CD9 is considered as important to anchor this protein to the lipid bilayer and to stabilize its transmembrane region (Umeda et al. 2020). Similar juxtamembrane cysteine residues of SPRI1 (C199, C200, C202) were found to be important for its full function (Fig. 7b) and for its stable accumulation (Fig. 7d), suggesting the commonality in the regulatory mechanism of SPRI1 and tetraspanins.

Relocation of intra and inter molecular disulfide bonds of the four transmembrane protein DsbB in *E. coli* has been reported and was proposed to regulate the function of this protein (Inaba et al. 2006). It is also possible that intra- and inter-molecular disulfide bond switching occur in SPRI1 and may regulate its function.

The protein complex of ∼300 kDa is formed by SPRI1, consisting of a mixture of intra-molecular linked SPRI1 monomer and inter-molecular linked SPRI1 dimers, suggesting that it is a SPRI1 homo-oligomer. Notably, this complex was also observed in C67A and C80A plants where almost all SPRI1 was inter-molecular bridged (Fig. 6b and 6c), indicating that the SPRI1-multimer contains an even number of SPRI1. One plausible function of this protein complex is acting as a molecular hub, where SPRI1 interacts with other proteins, such as tetraspanin proteins (Boucheix and Rubinstein 2001). It is possible that SPRI1 associates with other signaling components during pollination process. For example, receptor-like kinases such as FERONIA in Brassicaceae were found to be required to form interspecific reproductive barriers (Huang et al. 2023, Lan et al. 2023). The Brassicaceae self-incompatibility system triggers calcium signaling upon self-pollination (Iwano et al. 2015), which might also occur in rejecting heterospecific pollen grains. In some cases, four transmembrane proteins which form oligomeric structures are reported to function as ion or gap junction channels (Burendei et al., 2020, Collingridge et al. 2009). It may be possible that the oligomeric SPRI1 works as a channel. Given that SPRI1 has the ability to discriminate the species origin of pollen (Fujii et al. 2019), it is reasonable to assume that SPRI1 modulates the activity of its interacting partner(s) upon perceiving pollen grains.

## Materials and Methods

### Plant materials and growth condition

All non-transgenic plant materials were obtained as describe in the previous study (Fujii et al. 2019). All plant materials were grown in mixed soil in a growth chamber under controlled conditions (light intensity 120–150 µmol/m^2^/s, 14 h light/10 h dark cycle at 22 ± 2°C).

### Gene cloning, introduction of the mutations, deletions, and insertions

We used the *SPRI1A* genomic fragment carrying pCambia1300 binary plasmid (pCambia1300/*SPR1A* or pCambia1300/*SPRI1A-Venus*) created in our previous study (Fujii et al. 2019) as a template to introduce various mutations. We used a common QuikChange site-directed mutagenesis protocol mostly following the rationale in other studies (Liu and Naismith 2008). In brief, 1 ng of pCambia1300/*SPRI1A* was used as the template to perform the mutagenesis inverse PCR using KOD FX Neo polymerase kit (TOYOBO, Osaka, Japan). Inverse PCR in 50 µl reaction volume was performed with the following thermal routine: 98°C 30 s denature, followed by 12 cycles of 98°C 10 s; 55°C 5 s; 68°C 10 min. Primers are listed in Table S1. The restriction enzyme DpnI (Takara-Bio, Kusatsu, Japan) was added to the PCR product and the solution was incubated at 37°C for 1 h, to eliminate the template plasmid. The solution was then used to transform NEB turbo *E. coli* competent cells (NEB, MA, USA). pCambia1300/*SPRI1A-Ct+12* was created in our previous study (Fujii et al. 2019). The mutant plasmids were used to create the transgenic plants using the *Agrobacterium* infiltration procedure, as described in our previous study (Iwano et al. 2009).

### Pollination experiments

Flowers were emasculated before anthesis. At anthesis, pistils were harvested, placed on 1% agar plates, pollinated, and incubated for 6 h at 22 ± 2°C, humidity 50 ± 5%. Pistils were pollinated in a manner that entire stigmatic surface is covered with pollen grains. Pollinated pistils were fixed overnight at room temperature in ethanol/acetate 3:1 (v/v), then at 60°C for 30 min in 1 M sodium hydroxide. The pistils were stained in 2% tripotassium phosphate/0.01% aniline blue for 3 h at room temperature.

### Microscopic observation of pollen tubes

Pollen tubes on pistils stained with aniline blue were observed and counted under an epifluorescent microscope as previously described (Shiba et al. 2000). To facilitate large-scale phenotyping during the alanine scanning analysis, we defined arbitrary compatibility scores based on the numbers of pollen tubes in the styles: 1: no tubes observed; 2: 1–10 tubes; 3: 11–30 tubes; 4: 31–60 tubes; 5: ≥60 tubes.

### Microscopic observation of fluorescent proteins

Fluorescence in the emission range 520–555 nm was observed using a LSM880 confocal laser scanning microscope (Carl Zeiss, Oberkochen, Germany), with 514 nm excitation from Argon laser.

### Gene expression studies

Total RNA was extracted from stigmas using the RNeasy plant mini kit (Qiagen, Hilden, Germany) or a Maxwell 16 Instrument and Maxwell LEV Plant RNA Kit (Promega, WI, USA). Each kit includes a DNase I treatment. cDNA synthesis was done using the ReverTra Ace qPCR RT Master Mix (TOYOBO). The real-time PCR reaction was performed with the PowerUp SYBR Green Master Mix (Thermo Fischer Scientific, MA, USA) using the Applied Biosystems StepOne Real-Time PCR System (Thermo Fischer Scientific). All kits were used according to the manufacturer’s protocols. Primers are listed in Table S1.

### Procedures of obtaining antibodies against soluble parts of SPRI1

A synthetic gene-encoding SPRI1 of which transmembrane domains (39th–61st, 100th–122nd, 134th–156th, and 171st–193rd amino acids) were substituted by GGGGS linkers was purchased from FASMAC (Atsugi, Japan). This gene was amplified by PCR and inserted via In-Fusion system (TOYOBO) into the NdeI and XhoI sites of pET22b(+) (Novagen). Primers are listed in Table S1. Expression of the recombinant proteins was induced by 1 mM isopropyl β-d-thiogalactopyranoside at 37°C for 5 h in host *E. coli* strain Rosetta (DE3) pLysS cells (Novagen). After induction, the cells were harvested in 20 mM potassium phosphate buffer (pH 8.0) containing 500 mM NaCl and cOmplete EDTA-free protease inhibitor cocktail (Roche, Basel, Switzerland). The inclusion bodies were pelleted from sonicated cells at 3000 × *g* for 15 min and solubilized in 20 mM potassium phosphate buffer (pH 8.0) containing 500 mM NaCl and 6 M guanidine hydrochloride. Insoluble material was removed by centrifugation at 10000 × *g* for 1 h. The supernatant was incubated with COSMOGEL His-Accept (Nacalai, Kyoto, Japan) for 1 h. The beads were washed with 20 mM potassium phosphate buffer (pH 7.4) containing 500 mM NaCl and 4 M urea. The recombinant proteins were eluted with 20 mM potassium phosphate buffer (pH 7.4) containing 500 mM imidazole, 500 mM NaCl, and 4 M urea. Polyclonal antisera were raised against the purified recombinant protein in a mouse (T. K. Craft, Maebashi, Japan).

### Immunoblot analysis

Stigmas were collected from the *Arabidopsis* at flower stages (13–14) in 50 mM HEPES-KOH (pH 7.4) containing 5 mM MgCl_2_, 2 mM MnCl_2_, 10 mM NaF, 10 mM β-glycerophosphate, cOmplete ULTRA EDTA-free protease inhibitor cocktail (Roche), and 0.0125% (w/v) Tween-20 and stored at −80°C until use. One hundred stigmas were homogenized using a polypropylene pestle by hand in a 1.5 ml tube containing 100 µl of this buffer on ice. Debris was removed by 100 × *g* for 2 min, and supernatants were centrifuged at 20,000 × *g* for 30 min. Supernatants were collected as soluble fractions and used for detection of Actin. Remaining pellets were washed twice by resuspending in the 50 mM HEPES-KOH (pH 7.4) containing 5 mM MgCl_2_, 2 mM MnCl_2_, 10 mM NaF, 10 mM β-glycerophosphate, Complete ULTRA EDTA-free protease inhibitor cocktail and centrifugation (20,000 × *g*, 30 min). The final pellet was resuspended in 15 µl of the same buffer and used for detection of SPRI1A as a membrane fraction. After addition of equal amount of SDS sample buffer (50 mM Tris-HCl (pH 6.8) containing 2% SDS, and 10% (v/v) glycerol with or without 50 mM DTT), samples were incubated at 60°C for 15 min. For complete denaturation of the Venus structure, we performed trichloroacetic acid (TCA) precipitation in Fig. 3b. Protein samples solubilized by SDS were mixed well with TCA to a final concentration of 20% (w/v). After centrifugation at 20,000 × *g* for 5 min at 4°C, pellets were washed twice with cold acetone and solubilized again in the SDS sample buffer. TGX FastCast Acrylamide kit (12%, Bio-Rad, CA, USA) was used for SDS-PAGE. After electrophoresis, proteins were blotted on the Immobilon-P PVDF 0.45 µm (MERCK, Darmstadt, Germany). PVDF membranes were blocked using the PVDF Blocking Reagent for Can Get Signal (TOYOBO). Primary and secondary antibodies were diluted in the TBS-T buffer (0.05 M Tris-HCl (pH 7.6), 0.15 M NaCl, and 0.05% (v/v) Tween-20). Polyclonal antibody against Actin and GFP were purchased from Agrisera (AS13 2640, Vännäs, Sweden) and MBL (598, Tokyo, Japan), respectively, and used at a dilution of 1:5000. Polyclonal antibody against SPRI1 was used at a dilution of 1:2000. Secondary antibody against rabbit IgG [Goat Anti-Rabbit IgG (H + L)-HRP Conjugate (170–6515, Bio-Rad)] was used at a dilution of 1:25 000 for detection of Actin and Venus. Secondary antibody against mouse IgG [Goat Anti-Mouse IgG + IgM HRP conjugate (AMI0704, Biosource international, CA, USA)] was used at a dilution of 1:25,000 for detection of SPRI1.

### 2D BN/SDS-PAGE

Large pore BN-PAGE was performed as previously described (Järvi et al. 2011, Otani et al. 2018) with minor modifications. The pellet of membrane fractions prepared as described earlier were resuspended in 25 mM BisTris-HCl (pH 7.0) and centrifuged at 20,000 × *g* for 30 min. The pellet derived from 200 stigmas was resuspended in 10 µl of 25 mM BisTris-HCl containing 20% (w/v) glycerol and 1% (w/v) *n*-dodecyl-β-d-maltoside for 5 min on ice. Insoluble materials were removed by centrifugation at 20,000 × *g* for 2 min. Supernatants were mixed with one-tenth volume of 100 mM BisTris-HCl (pH 7.0) containing 500 mM 6-aminocaproic acid, 30% sucrose, and 5% SERVA Blue G, and membrane protein complexes equivalent to 200 stigmas were separated by 3% (T) 20% (C) acrylamide stacking gel and 3.5–12% (T) 3% (C) acrylamide gradient separating gel containing 50 mM BisTris-HCl (pH 7.0) and 500 mM 6-aminocaproic acid. NativeMark Unstained Protein Standard (Thermo Fischer Scientific, MA, USA) was also loaded. BN-gel was excised and incubated with SDS sample buffer [50 mM Tris-HCl (pH 6.8) containing 2% SDS, and 10% (v/v) glycerol with or without 50 mM DTT] for 15 min at 60°C. Gel strips were layered on TGX FastCast 12% Acrylamide gel. After electrophoresis, SPRI1 and Venus were detected as described above.

### Co-immunoprecipitation

The *ATML1* (*AT4G21750*) promoter, which specifically expresses at epidermis (Takada et al. 2013), and the *AtHSP18.2* (*AT5G59720*) terminator sequences were amplified from the Col-0 genomic DNA using PCR. We amplified the SPRI1A coding sequence from a previously constructed (Fujii et al. 2019) by PCR. The 5ʹ UTR of the *ADH* gene (*AT1G77120*) was fused to 5ʹ end of *SPRI1A*, and the DNA sequence encoding GGGSx3 linker and 2xHA was attached to 3ʹ end of *SPRI1A* using primers. *ATML1* promoter, *SPRI1A-2xHA*, and *AtHSP18.2* terminator sequences were fused in the second PCR. *SPRI1A-Venus* was amplified from the constructed plasmid described above with 5ʹ UTR of *ADH. ATML1* promoter, *SPRI1A-Venus*, and *AtHSP18.2* terminator sequences were fused in the second PCR. The resultant PCR products were inserted via In-Fusion system into the HindIII and *Sac*I sites of pCambia1300. Primers are listed in Table S1. The resultant plasmids were used for the transformation of *Agrobacterium tumefaciens* C58. Each transformed *Agrobacterium* was cultured in the LB medium at 28°C for 16 h and washed twice with 10 mM MES-KOH (pH 5.7) containing 10 mM MgCl_2_. Subsequently, the *Agrobacterium* was diluted tenfold with 10 mM MES-KOH (pH 5.7) containing 10 mM MgCl_2_ and 100 µM acetosyringone, and then infiltrated into the *Nicotiana benthamiana* leaves. Between 4 and 6 days post-infiltration, the leaves were used for the immunoprecipitation experiment. For the preparation, the leaves were homogenized in 50 mM HEPES-KOH (pH 7.4) containing 5 mM MgCl_2_, 2 mM MnCl_2_, and cOmplete EDTA-free protease inhibitor cocktail (Roche) using mortars and pestles. The debris were filtered using miracloth (Merck). The total membranes were pelleted by centrifugation (20 000 × *g*, 5 min). The pellet was washed twice with the same buffer, and the chlorophyll content was determined according to the previous method (Porra et al. 1989). The membrane fraction, equivalent to 400 µg of chlorophyll, was then washed twice with 50 mM Tris-HCl (pH 7.5) containing 150 mM NaCl and cOmplete EDTA-free protease inhibitor cocktail (Roche). Subsequently, the membrane protein complexes were solubilized in 500 µl of 50 mM Tris-HCl (pH 7.5) containing 150 mM NaCl, cOmplete EDTA-free protease inhibitor cocktail (Roche), and 1% (w/v) *n*-dodecyl-β-d-maltoside for 5 min on ice. Insoluble materials were removed by centrifugation at 20 000 × *g* for 5 min. The supernatants were mixed with a one-tenth volume of 10% A8-35 (Anatrace, OH, USA) and further centrifuged at 20 000 × *g* for 30 min. Aliquots of the supernatants were used as input samples. The remaining supernatants were incubated with 50 µl of µMACS Anti-GFP MicroBeads (Miltenyi Biotec, Bergisch Gladbach, Germany) and incubated on ice for 30 min. The beads were trapped in the µ Columns (Miltenyi Biotec) and washed four times with 50 mM Tris-HCl (pH 5.7) containing 150 mM NaCl, cOmplete EDTA-free protease inhibitor cocktail (Roche). The immunoprecipitated proteins were eluted with 1× SDS sample buffer [50 mM Tris-HCl (pH 6.8) containing 2% SDS, and 10% (v/v) glycerol]. SDS-PAGE and immunoblot analysis were carried out as described earlier.

### Sequence analysis and dN/dS calculation of *SPRI1* by PAML

TMHMM server v2.0 (Krogh et al. 2001) was used to predict the transmembrane region of SPRI1. Sequences of the *SPRI1* orthologous genes were obtained as described in our previous study (Fujii et al. 2019). Multiple alignment of the SPRI1 proteins were performed by muscle (Edgar 2004), followed by manual corrections (Supplementary Table S2). A codon-wise alignment of the *SPRI1* orthologs was performed using the tranalign function in the EMBOSS package. We used the site model in the Phylogenetic Analysis Using Maximum Likelihood (PAML) package to detect positively selected sites in the *SPRI1* orthologs. The dN/dS ratio was calculated with the two codon substitution models M7 and M8 included in the program codeml (Yang, 2007). M7 is a neutral model with continuous beta distribution of dN/dS values (≤1) is assumed. M8 overlaps with M7, except that it allows dN/dS > 1. M7 can be used as the null hypothesis against M8. All of the parameters in the codeml control file were default with ‘model’ set as 0. The fitness of codon substitution models was evaluated with log-likelihood ratio statistics, which are assumed to be χ^2^ distributed. Bayes Empirical Bayes (BEB) probabilities of positive selection and dN/dS values for each site were found from the rst output file from the codeml run.

### Statistical analysis

All of the statistical analysis was done using R (R Core Team 2017). For all box plots, center lines show the medians, box limits indicate the 25th and 75th percentiles, whiskers extend 1.5 times the interquartile range from the 25th and 75th percentiles, and data points are plotted as open circles.

## Supplementary Material

pcaf039_Supp

## Data Availability

The data that support the findings of this study are available on request.

## References

[R1] Boucheix C. and Rubinstein E. (2001) Tetraspanins. *Cell. Mol. Life. Sci*. 58: 1189–1205. doi: 10.1007/PL00000933 11577978 PMC11337403

[R2] Burendei B., Shinozaki R., Watanabe M., Terada T., Tani K., Fujiyoshi Y., et al. (2020) Cryo-EM structures of undocked innexin-6 hemichannels in phospholipids. *Sci. Adv*. 6: 1–10. doi: 10.1126/sciadv.aax3157 PMC7015682 32095518

[R3] Charrin S., Jouannet S., Boucheix C. and Rubinstein E. (2014) Tetraspanins at a glance. *J. Cell. Sci*. 127: 3641–3648. doi: 10.1242/jcs.154906 25128561

[R4] Charrin S., Manié S., Oualid M., Billard M., Boucheix C. and Rubinstein E. (2002) Differential stability of tetraspanin/tetraspanin interactions: role of palmitoylation. *FEBS Lett*. 516: 139–144. doi: 10.1016/S0014-5793(02)02522-X 11959120

[R5] Collingridge G.L., Olsen R.W., Peters J. and Spedding M. (2009) A nomenclature for ligand-gated ion channels. *Neuropharmacology*. 56: 2–5. doi: 10.1016/j.neuropharm.2008.06.063 18655795 PMC2847504

[R6] de Nettancourt D. (2001) Incompatibility and Incongruity in Wild and Cultivated Plants. Springer, Berlin, Heidelberg.

[R7] Edgar R.C. (2004) MUSCLE: multiple sequence alignment with high accuracy and high throughput. *Nucleic. Acids. Res*. 32: 1792–1797. doi: 10.1093/nar/gkh340 15034147 PMC390337

[R8] Fujii S., Tsuchimatsu T., Kimura Y., Ishida S., Tangpranomkorn S., Shimosato-Asano H., et al. (2019) A stigmatic gene confers interspecies incompatibility in the Brassicaceae. *Nat. Plants*. 5: 731–741. doi: 10.1038/s41477-019-0444-6 31263241

[R9] Fujii S., Yamamoto E., Ito S., Tangpranomkorn S., Kimura Y., Miura H., et al. (2023) SHI family transcription factors regulate an interspecific barrier. *Nat. Plants* 9: 1862–1873. doi: 10.1038/s41477-023-01535-5 37798337

[R10] Gorleku O.A., Barns A.-M., Prescott G.R., Greaves J. and Chamberlain L.H. (2011) Endoplasmic reticulum localization of DHHC palmitoyltransferases mediated by lysine-based sorting signals. *J. Biol. Chem*. 286: 39573–39584. doi: 10.1074/jbc.M111.272369 21926431 PMC3234780

[R11] Huang J., Yang L., Yang L., Wu X., Cui X., Zhang L., et al. (2023) Stigma receptors control intraspecies and interspecies barriers in Brassicaceae. *Nature*. 614: 303–308. doi: 10.1038/s41586-022-05640-x 36697825 PMC9908550

[R12] Inaba K. and Ito K. (2008) Structure and mechanisms of the DsbB–DsbA disulfide bond generation machine. *Biochim. Biophys. Acta. Mol. Cell Res*. 1783: 520–529. doi: 10.1016/j.bbamcr.2007.11.006 18082634

[R13] Inaba K., Murakami S., Suzuki M., Nakagawa A., Yamashita E., Okada K., et al. (2006) Crystal structure of the DsbB-DsbA complex reveals a mechanism of disulfide bond generation. *Cell*. 127: 789–801. doi: 10.1016/j.cell.2006.10.034 17110337

[R14] Iwano M., Entani T., Shiba H., Kakita M., Nagai T., Mizuno H., et al. (2009) Fine-tuning of the cytoplasmic Ca2+ concentration is essential for pollen tube growth. *Plant. Physiol*. 150: 1322–1334. doi: 10.1104/pp.109.139329 19474213 PMC2705041

[R15] Iwano M., Ito K., Fujii S., Kakita M., Asano-Shimosato H., Igarashi M., et al. (2015) Calcium signalling mediates self-incompatibility response in the Brassicaceae. *Nat. Plants*. 1: 15128. doi: 10.1038/nplants.2015.128 27250681

[R16] Järvi S., Suorsa M., Paakkarinen V. and Aro E.-M. (2011) Optimized native gel systems for separation of thylakoid protein complexes: novel super- and mega-complexes. *Biochem. J*.. 439: 207–214. doi: 10.1042/BJ20102155 21707535

[R17] Kovalenko O.V., Yang X., Kolesnikova T.V. and Hemler M.E. (2004) Evidence for specific tetraspanin homodimers: inhibition of palmitoylation makes cysteine residues available for cross-linking. *Biochem. J*. 377: 407–417. doi: 10.1042/bj20031037 14556650 PMC1223880

[R18] Krogh A., Larsson B., von Heijne G. and Sonnhammer E.L. (2001) Predicting transmembrane protein topology with a hidden Markov model: application to complete genomes. *J. Mol. Biol*.. 305: 567–580. doi: 10.1006/jmbi.2000.4315 11152613

[R19] Lan Z., Song Z., Wang Z., Li L., Liu Y., Zhi S., et al. (2023) Antagonistic RALF peptides control an intergeneric hybridization barrier on Brassicaceae stigmas. *Cell*. 186: 4773–4787.e12. doi: 10.1016/j.cell.2023.09.003 37806310 PMC10615786

[R20] Lee H.K., Canales Sanchez L.E., Bordeleau S.J. and Goring D.R. (2024) Arabidopsis leucine-rich repeat malectin receptor–like kinases regulate pollen–stigma interactions. *Plant Physiol*. 195: 343–355. doi: 10.1093/plphys/kiae038 38270530

[R21] Liu H. and Naismith J.H. (2008) An efficient one-step site-directed deletion, insertion, single and multiple-site plasmid mutagenesis protocol. *BMC Biotechnol*. 8: 91. doi: 10.1186/1472-6750-8-91 PMC2629768 19055817

[R22] Morrison K.L. and Weiss G.A. (2001) Combinatorial alanine-scanning. *Curr. Opin. Chem. Biol*. 5: 302–307. doi: 10.1016/S1367-5931(00)00206-4 11479122

[R23] Nagai T., Ibata K., Park E.S., Kubota M., Mikoshiba K. and Miyawaki A. (2002) A variant of yellow fluorescent protein with fast and efficient maturation for cell-biological applications. *Nat. Biotechnol*. 20: 87–90. doi: 10.1038/nbt0102-87 11753368

[R24] Otani T., Kato Y. and Shikanai T.. (2018) Specific substitutions of light-harvesting complex I proteins associated with photosystem I are required for supercomplex formation with chloroplast NADH dehydrogenase-like complex. *Plant J*. 94: 122–130. doi: 10.1111/tpj.13846 29385648

[R25] Porra R.J., Thompson W.A. and Kriedemann P.E. (1989) Determination of accurate extinction coefficients and simultaneous equations for assaying chlorophylls *a* and *b* extracted with four different solvents: verification of the concentration of chlorophyll standards by atomic absorption spectroscopy. *Biochim. Biophys. Acta. Bioenerg*. 975: 384–394. doi: 10.1016/S0005-2728(89)80347-0

[R26] R Core Team (2017).

[R27] Ren J., Wen L., Gao X., Jin C., Xue Y. and Yao X. (2008) CSS-Palm 2.0: an updated software for palmitoylation sites prediction. *Protein Eng. Des. Sel*. 21: 639–644. doi: 10.1093/protein/gzn039 18753194 PMC2569006

[R28] Shiba H., Kimura N., Takayama S., Hinata K., Suzuki A. and Isogai A. (2000) Alteration of the self-incompatibility phenotype in Brassica by transformation of the antisense SLG gene. *Bio. Sci. Biotechnol. Biochem*. 64: 1016–1024. doi: 10.1271/bbb.64.1016 10879472

[R29] Takada S., Takada N. and Yoshida A. (2013) ATML1 promotes epidermal cell differentiation in Arabidopsis shoots. *Development*. 140: 1919–1923. doi: 10.1242/dev.094417 23515472

[R30] Takeuchi H. and Higashiyama T. (2016) Tip-localized receptors control pollen tube growth and LURE sensing in Arabidopsis. *Nature*. 531: 245–248. doi: 10.1038/nature17413 26961657

[R31] Trenevska I., Anderson A.P., Bentley C., Hassanali T., Wiblin S., Maguire S., et al. (2021) Comprehensive mutagenesis identifies the peptide repertoire of a p53 T-cell receptor mimic antibody that displays no toxicity in mice transgenic for human HLA-A*0201. *PLoS One*. 16: e0249967. doi: 10.1371/journal.pone.0249967 PMC8034716 33836029

[R32] Tsuchimatsu T. and Fujii S.. (2022) The selfing syndrome and beyond: diverse evolutionary consequences of mating system transitions in plants. *Philos. Trans. R. Soc. B. Biol. Sci*. 377: 1855 doi: 10.1098/rstb.2020.0510 PMC9149797 35634918

[R33] Umeda R., Nishizawa T. and Nureki O.. (2019) Crystallization of the human tetraspanin protein CD9. *Acta. Crystallogr. Sect. F. Struct. Biol. Commun*. 75: 254–259. doi: 10.1107/S2053230X1801840X 30950826 PMC6450527

[R34] Umeda R., Satouh Y., Takemoto M., Nakada-Nakura Y., Liu K., Yokoyama T., et al. (2020) Structural insights into tetraspanin CD9 function. *Nat. Commun*.. 11: 1–7. doi: 10.1038/s41467-020-15459-7 32231207 PMC7105497

[R35] Yang Z . (2007) PAML 4: phylogenetic analysis by maximum likelihood. *Mol. Biol. Evol*. 24: 1586–1591. doi: 10.1093/molbev/msm088 17483113

