# Supplementary material for: In-depth amino acid mutational analysis of the key interspecific incompatibility factor Stigmatic Privacy 1
Source: Plant Cell Physiol. 2025 Apr 15;66(6):926–39. doi: 10.1093/pcp/pcaf039 (PMC12290284; doi:10.1093/pcp/pcaf039)
Supplement: pcaf039_Supp [file pcaf039_supp.zip › suppl_data/pcp-2025-e-00032-File009.pdf]

Supplementary Figure S1

A

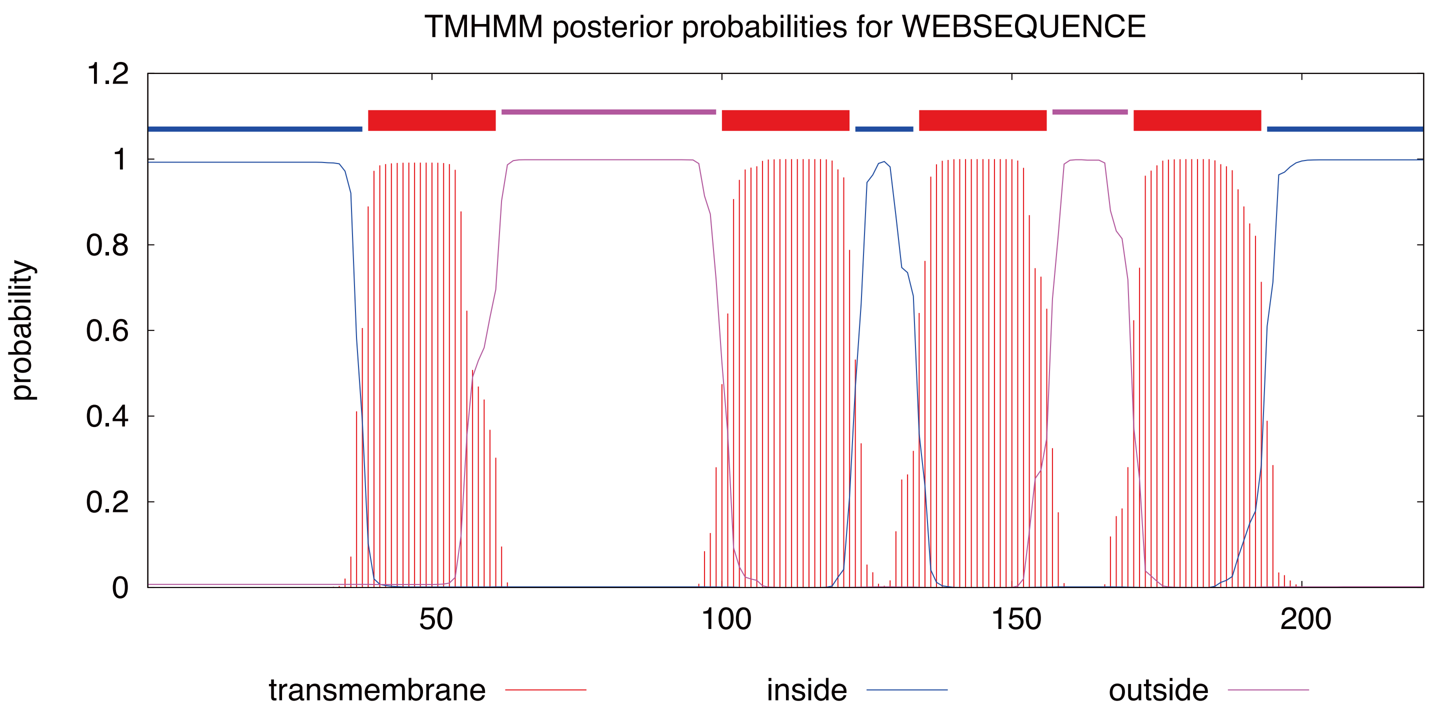

B

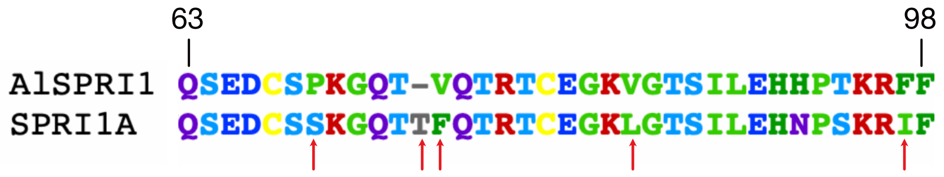

Supplementary Figure 1
(A) A prediction result of the transmembrane regions of SPRI1 using TMHMM. **(B)** The amino acid sequence alignment of SPRI1 in *A. lyrata* (AlSPRI1) and SPRI1A (Q63 to F98). The red arrows indicate amino acids not conserved between two sequences.

Supplementary Figure S2

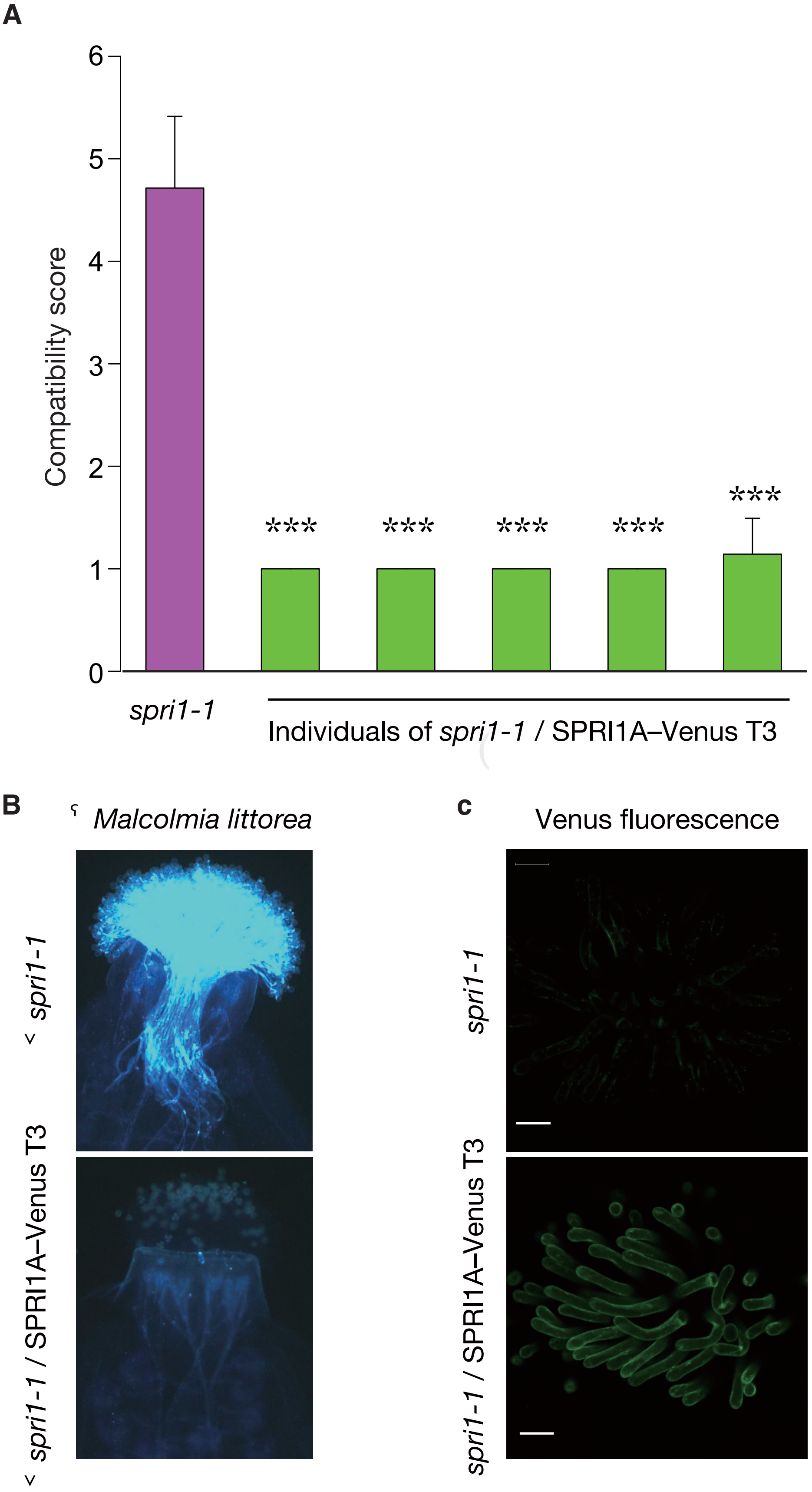

Supplementary Figure 2.
(A) The interspecific pollination assay in *spri1-1* and *spri1-1/SPRI1A-Venus*. Significant differences by Dunnett's test compared against *spri1-1* are indicated by ***($p < 0.005$). $n \geq 5$ pistils each. **(B)** Representative images of the interspecific pollination assay after aniline blue staining. **(C)** The expression of Venus-fused SPRI1A at stigma was confirmed at stigmatic papilla cells in *spri1-1/SPRI1A-Venus*. *spri1-1* was observed as a negative control.

Supplementary Figure S3

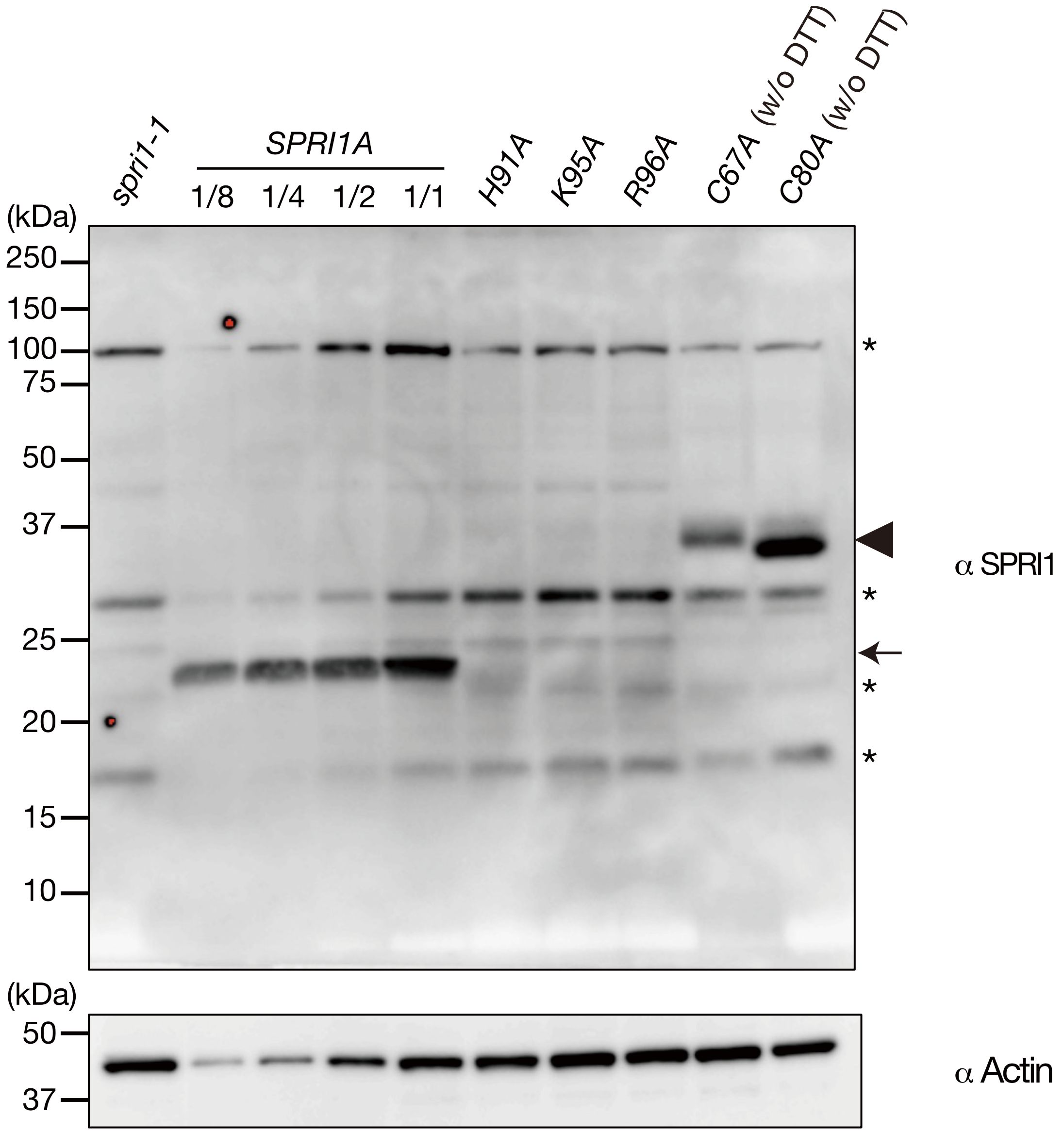

Supplementary Figure 3.

Total membrane proteins isolated from stigma samples of *SPRI1A*, *spri1-1*, and the lines expressing the alanine-replaced SPRI1A (*H91A*, *K95A*, *R96A*, *C67A*, *C80A*) were analyzed by immunoblotting using antibody against SPRI1. Because of the destabilization of SPRI1A_C67A and SPRI1A_C80A after addition of the SDS sample buffer including DTT, these samples were analyzed in the non-reducing condition. Sample loading was based on the number of stigmas, along with a dilution series of *SPRI1A*. Using total soluble proteins, actin was detected as a loading control with its specific antibody. A black arrow and an arrowhead indicate the SPRI1 monomer and the putative SPRI1 dimer, respectively. Black asterisks indicate non-specific signal.

Supplementary Figure S4

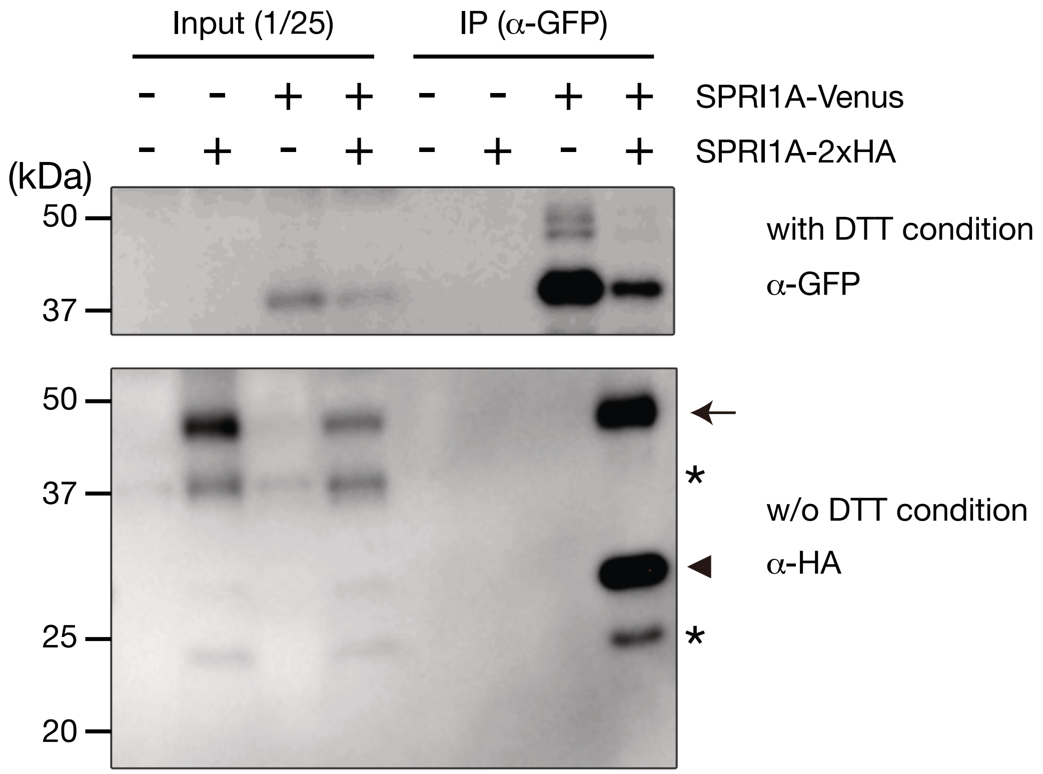

Supplementary Figure 4.

Co-immunoprecipitation analysis of SPRI1A-Venus and SPRI1A-2xHA expressed transiently in *Nicotiana benthamiana*. Solubilized total membrane proteins were immunoprecipitated using magnetic beads conjugated with the antibody against GFP. Input and immunoprecipitated (IP) samples were analyzed by SDS-PAGE and immunoblotting. For the detection of SPRI1A-2xHA, SDS-PAGE was performed under the non-reducing condition, whereas DTT was supplied for SPRI1A-Venus detection. The arrowhead and arrow indicate SPRI1A-2xHA monomer and putative dimer, respectively. The asterisks indicate possible degraded products of SPRI1A-2xHA. It was observed that the migration of the SPRI1A-Venus monomer was faster than in Figure 3B because TCA treatment was not performed in this experiment, and Venus was not fully denatured.

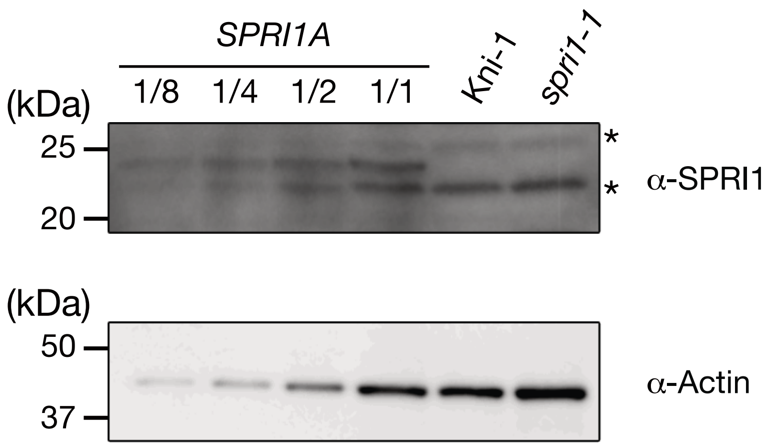

Supplementary Figure 5.

Total membrane proteins isolated from stigma samples of *SPRI1A*, Kni-1, and *spri1-1* were analyzed by immunoblotting using antibody against SPRI1. Sample loading was based on the number of stigmas, along with a dilution series of *SPRI1A*. Using total soluble proteins, actin was detected as a loading control with its specific antibody. The asterisks indicate non-specific signals.

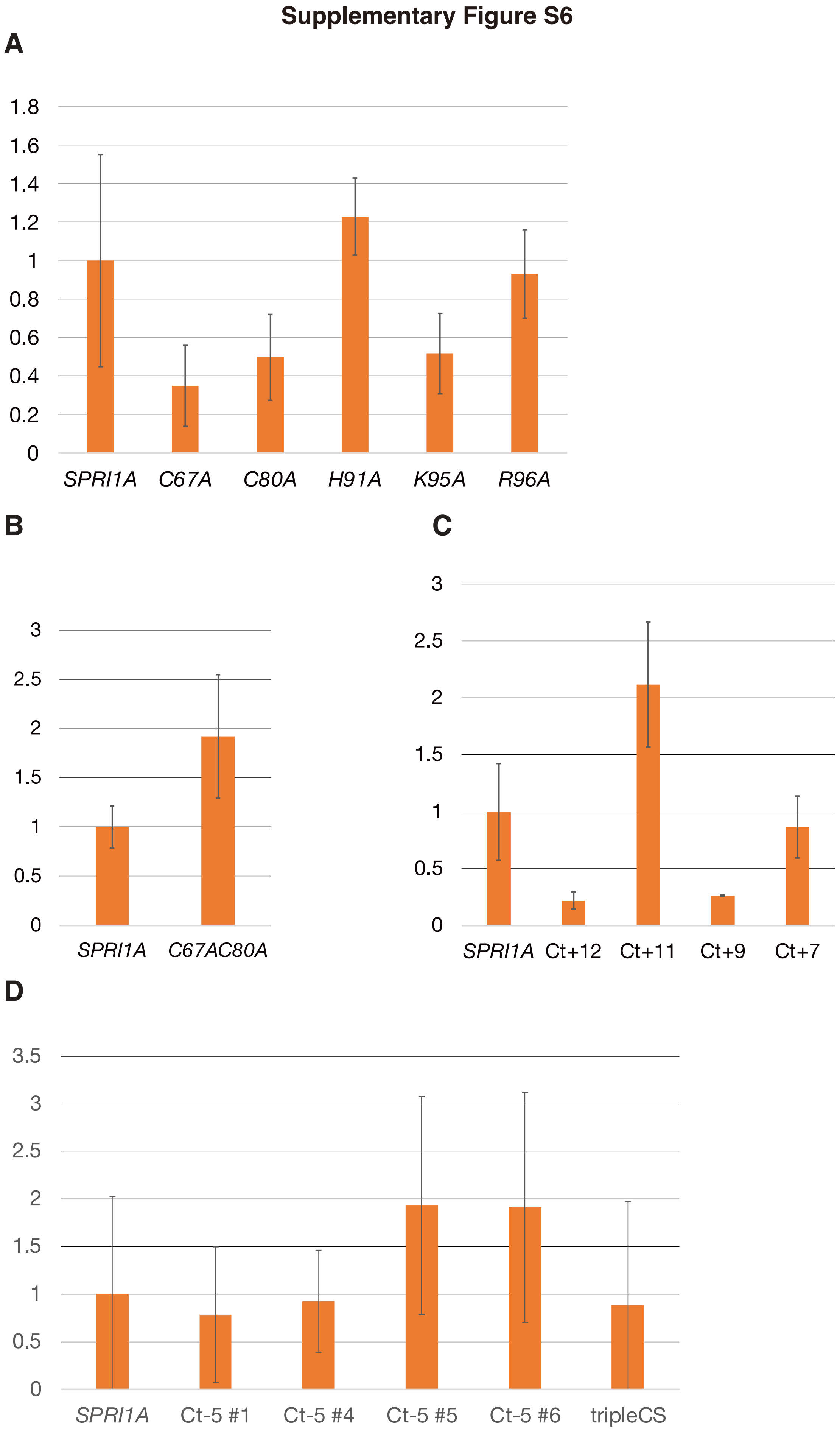

Supplementary Figure 6.

(A–D) Relative mRNA expression levels of SPRI1 compared to *Act8* in stigmatic tissues of the indicated lines. Values indicate means of three biological replicates and whiskers indicate standard deviations.

Supplementary Figure S7

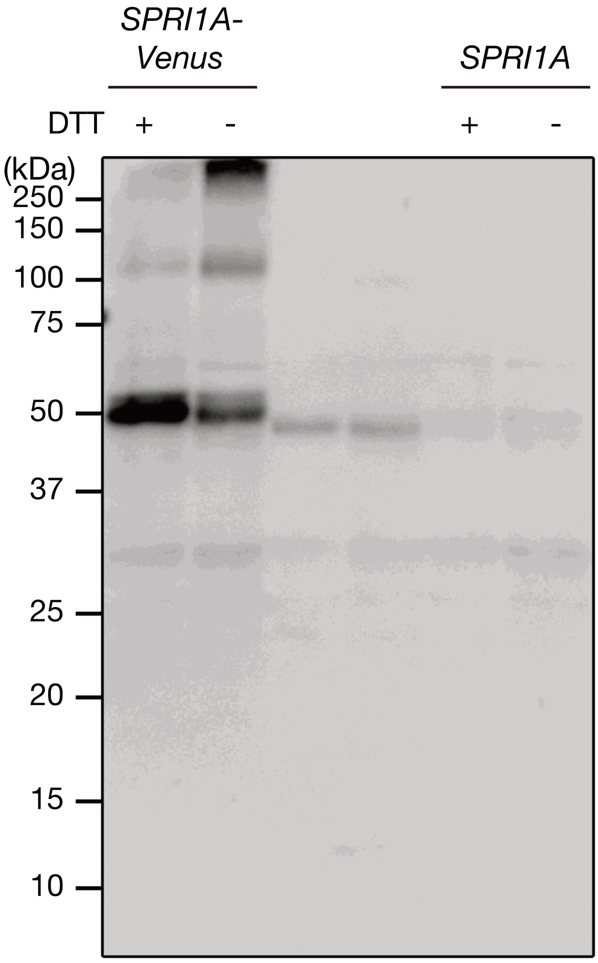

Supplementary Figure 7.

The uncropped image of Figure 3B.
